# A spatially defined Sr/Zn gradient coating with dual osteogenic and anti-corrosion functions on Ti6Al4V *via* softened spark MAO

**DOI:** 10.1039/d5ra08533k

**Published:** 2026-01-27

**Authors:** Yiting Hao, Liwei Ning, Jun Fu, Xiaoming Wang, Zixiong Zhou

**Affiliations:** a College of Mechanical and Energy Engineering, Shaoyang University Shaoyang 422000 China 18873994427@163.com 919513838@qq.com

## Abstract

In order to overcome the problems of highly interconnected pores and insufficient long-term corrosion resistance in micro-arc oxidation (MAO) coatings this study integrated softened spark MAO (S-MAO) with a Sr^2+^/Zn^2+^ complexation regulation strategy to fabricate composite MAO coatings on Ti6Al4V implant surfaces. The S-MAO treatment transformed coating architecture from a bilayer into a three-layer gradient structure (barrier layer/softened-spark layer/porous layer). This significantly diminished the interconnectedness of interior pores. Electrochemical tests showed that the way S-MAO coatings corrode changed from diffusion control to the interfacial charge transfer process being the most important. This indicates that the coatings' electrochemically protective qualities are more stable. The Z_1_S_3_MAO coating, with a Sr/Zn ratio of 3 : 1, demonstrated elevated interfacial charge transfer resistance (443.6 Ω cm^2^) and enhanced impedance responsiveness. The examination of the elemental distribution showed that Sr was spread out evenly throughout the coating thickness. However, Zn had a localized enrichment zone at the interface between the softened-spark layer and the porous outer layer. The spatial distribution is determined by the varying stability of ion complexation. The Z_1_S_3_MAO coating exhibited superior performance regarding wettability and cytocompatibility associated with osteogenesis.

## Abstract 2

Fabrication process of a spatially defined Sr/Zn gradient coating on Ti6Al4V by softened-spark MAO.

## Graphical abstract

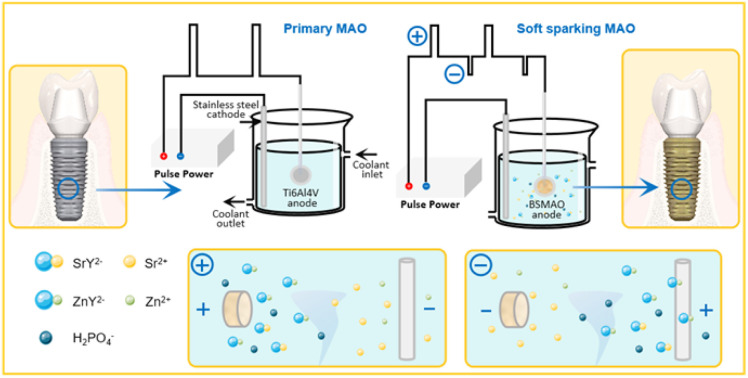

## 1. Introduction

Tooth loss severely impairs chewing efficiency and reduces quality of life. It is also linked to higher risks of systemic diseases, including stroke and cardiovascular disease, through impacts on nutrition and inflammation.^1,2^ Research further indicates that severe tooth loss is an independent risk factor for all-cause mortality, thereby directly impacting an individual's lifespan.^3^ To address this serious health issue, dental implants have become the primary clinical solution for restoring dental function and oral health.^4,5^ Titanium and its alloys, especially Ti6Al4V, are the material of choice for implants due to their excellent mechanical strength and biocompatibility.^6^ However, within the complex oral electrolyte environment, titanium-based implants remain susceptible to localized corrosion and mechanical wear. This process may lead to release metal ions or wear debris, thereby inducing local inflammatory responses and bone resorption, which may lead to aseptic loosening and even implant failure.^7,8^ These challenges impose higher requirements on the corrosion resistance and osseointegration stability of implant surfaces.

Surface modification techniques, including plasma spraying, sandblasting and acid etching, hydrothermal treatment, sol–gel processing, and MAO, have been established as effective approaches for enhancing long-term stability of titanium alloy implants.^9,10^ Among these, MAO has emerged as a current research focus, owing to advantages such as process maturity, environmental friendliness, and excellent coating performance.^11^ The MAO technique utilizes plasma electrolytic deposition to *in situ* fabricate porous ceramic layers on titanium substrates with high bonding strength.^12^ The resulting micro-nano porous structure facilitates simulation of extracellular matrix environments, thereby promoting cell adhesion and bone tissue ingrowth.^10,13^ However, the high-temperature discharges inherent to MAO inevitably introduce numerous micropores and microcracks within coatings.^14,15^ These interconnected defects provide rapid penetration channels for corrosive media. Consequently, the long-term protective function of the inner barrier layer is compromised, which can accelerate localized corrosion processes.^16,17^

Recently, softened spark micro-arc oxidation (S-MAO) has attracted broad attention as an effective approach for optimizing MAO coating structure. By adjusting the charge ratio between the cathode and anode and reducing discharge intensity, the softened spark mode promotes *in situ* growth of a dense inner layer at the coating/substrate interface. This process significantly enhances coating densification and electrochemical stability while preserving the outer micro-nano porous structure.^18,19^ Moreover, this unique interfacial nucleation-growth mechanism provides a more stable and reproducible deposition environment for controlled incorporation of multiple functional elements into coatings.^20,21^ To further enhance the biofunctionality of MAO coatings, bioactive metal ions such as strontium (Sr) and zinc (Zn) are widely introduced into electrolytes. Existing studies indicate that Sr^2+^ can significantly promote osteoblast activity and inhibit bone resorption, thereby effectively accelerating osseointegration.^22,23^ Zn^2+^ plays important roles in cellular metabolism and exhibits certain antibacterial activity.^24^ Consequently, Sr/Zn co-doped coatings are regarded as a surface modification strategy capable of combining osteogenic promotion with antibacterial function. In fact, studies have shown that such coatings exhibit positive effects on survival and mineralization of osteogenic cells, as well as antibacterial performance.^25–27^

However, literature review reveals that most current research primarily adjusts coating performance by altering absolute addition amounts of single metal ions. In multi-component MAO electrolyte systems, migration, deposition, and incorporation behaviors of different metal ions are often co-influenced by the relative content, ionic size, and competitive processes.^28^ Using absolute concentration as the sole variable fails to comprehensively reflect actual doping behavior under multi-ion coexistence. Particularly within the relatively stable growth environment of S-MAO, varying Sr/Zn ratios may influence ion migration kinetics and interfacial deposition behavior. This, in turn, regulates coating structural evolution, elemental spatial distribution, synergistic corrosion resistance and biological performance. Under conditions of fixed total metal ion concentration, systematic investigation into the effects of the Sr/Zn ratio on the structure and properties of composite MAO coatings remains insufficient.

**Fig. 1:**
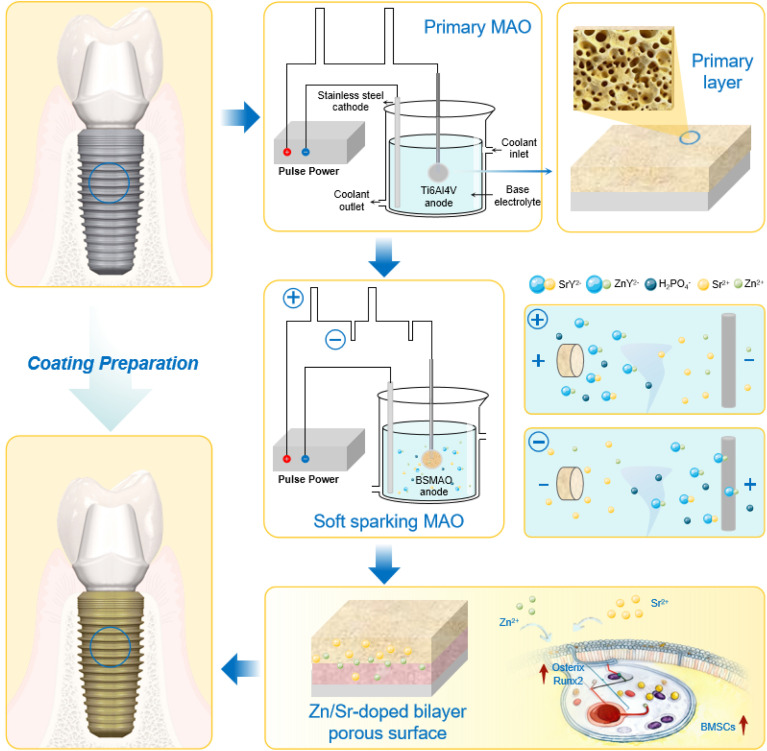
Schematic illustration of the fabrication process for Sr/Zn co-doped gradient coatings on Ti6Al4V dental implant surfaces.

To address these limitations, this study introduces S-MAO combined with a disodium ethylenediaminetetraacetate (EDTA-2Na) complexation regulation strategy. By maintaining constant total metal ion concentration, the Sr^2+^/Zn^2+^ ratio in the electrolyte was systematically adjusted to establish a multi-ion MAO preparation system with controllable ion ratios. The specific process flow is shown in Fig. 1. Using this system, composite MAO coatings with different Sr/Zn ratios were fabricated on Ti6Al4V substrates, and the structural changes and elemental distribution characteristics were systematically characterized. Further analysis integrating electrochemical and biological performance was conducted to better understand the role of Sr/Zn ratio variation in coating structure and stability. The resulting insights are expected to support the development of multifunctional surface designs for titanium alloy implants.

## 2. Preparation of MAO coatings

### 2.1. Substrate preparation

Ti6Al4V discs (*Φ*12 mm × 6 mm, Taikewoo Titanium Co., Ltd, Guangzhou, China) were used as substrate materials, and the chemical composition is listed in Table 1. An M3 threaded hole was machined on the lateral surface of each disc to enable connection to the suspended electrode holder during MAO processing. Prior to surface treatment, all specimens were sequentially ground using silicon carbide (SiC) abrasive papers with grit sizes of 120, 800, 1200, and 2000, followed by polishing with 1.0 µm diamond suspension for a smooth, uniform finish. The polished samples were ultrasonically cleaned in acetone and ethanol for 10 min each, and then dried at room temperature for subsequent experiments.

**Table 1:** Chemical composition of Ti6Al4V (wt%)

Ti	Al	V	Fe	O	H
89.285%	6.100%	4.100%	0.300%	0.200%	0.015%

### 2.2. Coating preparation

#### 2.2.1. Initial coating preparation

For MAO processing, cleaned specimens served as the anode, with the stainless-steel rod (*Φ*5 mm × 30 mm) as the cathode at a fixed inter-electrode distance of 15 cm. A bipolar square-wave pulse power supply (Model AN7505, Annaisi Electronics Technology Co., Ltd, Wuxi, China) was operated in constant-current mode for two-stage fabrication of the gradient coating. Based on prior research, the first stage employed single-pulse MAO, which was performed in the electrolyte containing 0.09 mol L^−1^ Na_2_B_4_O_7_·10H_2_O and 0.01 mol L^−1^ Na_2_SiO_3_·9H_2_O (BS electrolyte) to deposit an initial coating with minimal cracks and favorable properties.^29^ Process parameters were set at 10% duty cycle, 200 Hz frequency, and 0.45 A current for 480 s. Specimens obtained from this step were designated BSMAO.

#### 2.2.2. Composite coatings preparation

For S-MAO processing, BSMAO specimens served as the anode and a stainless-steel rod as the cathode. Electrolyte temperature was maintained at 10 ± 2 °C using a circulation cooler. Phosphate-based electrolytes were prepared by adding zinc acetate dihydrate (C_4_H_6_O_4_Zn⋯2H_2_O) and strontium (C_4_H_6_O_4_Sr) acetate as sources of Zn^2+^ and Sr^2+^ ions, with EDTA-2Na serving as chelating agent. The total metal ion (Sr^2+^ + Zn^2+^) molar concentration was kept constant, and four Sr^2+^ proportion gradients were designed: 87.5%, 75%, 50%, and 25%. Detailed electrolyte compositions, conductivity, and pH are listed in Table 2. All reagents (analytical grade) were supplied by Sinopharm Chemical Reagent Co., Ltd. The softened spark treatment lasted 600 s with following electrical parameters: anode and cathode duty cycles both at 16.7%, anode current at 0.164 A, cathode current at 0.45 A, and operating frequency at 120 Hz. Resulting specimens were ultrasonically cleaned in absolute ethanol, vacuum-dried at 40 °C, and designated as Z_1_S_7_MAO, Z_1_S_3_MAO, Z_1_S_1_MAO, and Z_3_S_1_MAO according to the Sr/Zn molar ratio in respective electrolytes.

**Table 2:** Electrolyte composition used in the preparation of the softened sparking layer

Samples	NH_4_H_2_PO_4_	EDTA-2Na	Sr(C_2_H_3_O_2_)_2_	Zn(C_2_H_3_O_2_)_2_·2H_2_O	Conductivity
Z_1_S_7_MAO	0.12 mol L^−1^	0.20 mol L^−1^	0.175 mol L^−1^	0.025 mol L^−1^	24.0 S m^−1^
Z_1_S_3_MAO	0.12 mol L^−1^	0.20 mol L^−1^	0.15 mol L^−1^	0.05 mol L^−1^	24.8 S m^−1^
Z_1_S_1_MAO	0.12 mol L^−1^	0.20 mol L^−1^	0.10 mol L^−1^	0.10 mol L^−1^	26.1 S m^−1^
Z_3_S_1_MAO	0.12 mol L^−1^	0.20 mol L^−1^	0.05 mol L^−1^	0.15 mol L^−1^	26.9 S m^−1^

### 2.3. Coating characterization and testing

#### 2.3.1. Stability of the coating preparation process

**Fig. 2:**
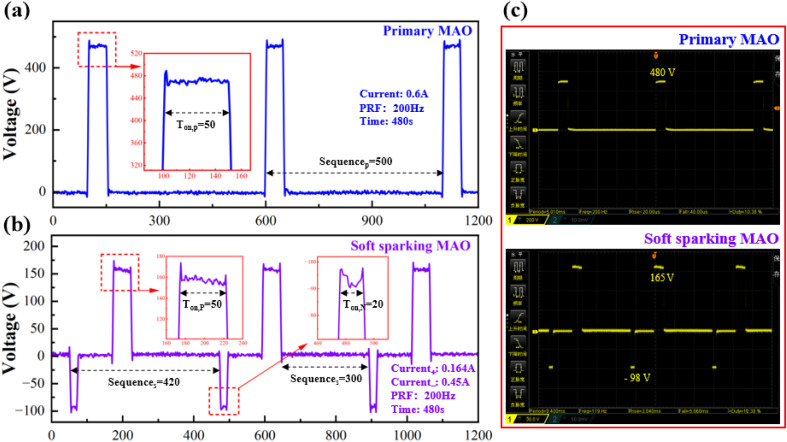
Voltage waveforms recorded during the MAO process: (a) initial single-pulse MAO; (b) softened spark MAO; (c) oscilloscope waveforms of the two-step MAO process.

During the coating preparation process, waveform curves were recorded at 200-second intervals using an oscilloscope (RIGOL DS1102Z-E, China), as shown in Fig. 2. In the MAO process, the actual frequency was maintained at 200 Hz with a duty cycle of approximately 10.0% (Fig. 2(a)). Under the S-MAO conditions, the operating frequency was approximately 117.4 Hz, with the positive and negative duty cycles being approximately 11.7% and 6.3%, respectively (Fig. 2(b)). The actual frequency and duty cycle of both processes were in close agreement with the preset values, indicating that the changes in electrolyte composition and electrical parameters during the coating preparation process had no significant effect on the process. The oscilloscope's real-time interface is shown in Fig. 2(c), where the voltage curve exhibits stable fluctuations, and the experiment proceeded smoothly overall.

#### 2.3.2. Analysis of coating structure and physicochemical properties

Phase composition of the coating surface was analyzed using X-ray diffraction (XRD) with Cu Kα radiation (AL2700B, China) at an operating voltage of 60 kV and current of 60 mA. The diffraction angle (2*θ*) was scanned from 5° to 85° at a rate of 2°/min. Surface morphology and cross-sectional structure of the samples were examined using a focused ion beam scanning electron microscope (ZEISS Sigma 300, Germany). Energy dispersive spectroscopy (EDS) analysis (Xplore 30, UK) was employed to characterize the distribution of Sr, Zn, phosphorus (P), titanium (Ti), oxygen (O), and silicon (Si) on the surface and cross-section of the coating. The porosity and pore size of the coating were quantified using Image-Pro Plus 5.0 software. The 3D topography and surface roughness of finished samples were detected using a Laser Scanning Confocal Microscope (OLS4000, Olympus Corporation, Japan), with a data collection interval of 0.5 s. The surface wettability of the coatings was characterized using an optical contact angle goniometer (Attension Theta Flex, Biolin Scientific AB, Sweden).

#### 2.3.3. Electrochemical performance analysis of the coatings

Electrochemical tests, including polarization and electrochemical impedance spectroscopy (EIS), were performed using three-electrode electrochemical system (CHI660E, CHI Instruments Co., Ltd, Shanghai, China). Saturated calomel electrode (SCE) was used as the reference electrode, and platinum electrode was used as the counter electrode. The sample was connected to conductive copper rod as working electrode, with fixed exposed surface area of 1 cm^2^. Prior to testing, open circuit potential (OCP) was measured for 400 s to allow stabilization. Electrochemical corrosion behavior of the samples was evaluated in 0.01 M PBS buffer solution at pH 7.2. The polarization potential range was set from ±350 mV (*vs.* SCE) relative to OCP, with scan rate of 0.01 V s^−1^. EIS measurements were conducted under same electrolyte conditions, with frequency range from 10 mHz to 100 kHz. Obtained EIS data were analyzed using ZSimpWin software (v.3.60, EChem Software, USA), and impedance response behavior of the coatings was interpreted by selecting an appropriate equivalent circuit model.

#### 2.3.4. Cell biocompatibility evaluation

The *in vitro* biocompatibility of the dental implants was evaluated by performing LIVE/DEAD staining (Live/Dead assay) on MC3T3-E1 murine pre-osteoblast cells. The cells were cultured under standard conditions (37 °C, 5% CO_2_) for 24 hours. After the incubation period, the cells were stained with a working solution containing reagent A (calcein-AM) and reagent B (Propidium Iodide, PI). After staining, the samples were washed three times with PBS and observed under a confocal laser scanning microscope (LSM 900, Carl Zeiss, Germany) to capture fluorescent images.

## 3. Results and discussion

### 3.1. Voltage-time response characteristics

**Fig. 3:**
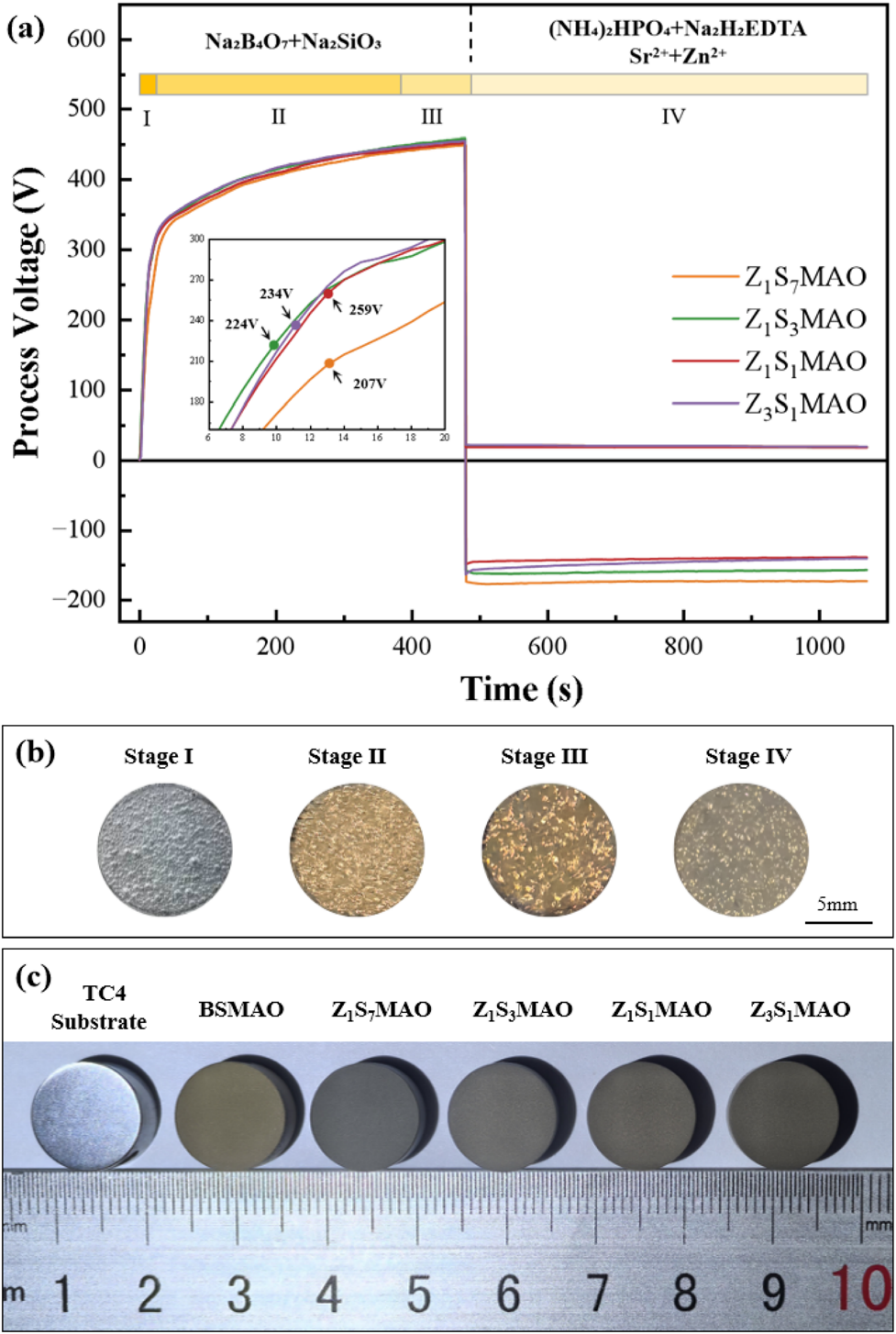
The treated MAO titanium alloy in different electrolytes: (a) voltage-time response curves; (b) spark morphologies at various stages; (c) sample surface morphologies.

Fig. 3(a and b) presents voltage-time response curves and spark discharge morphology during two-step MAO processing. The MAO process comprised three characteristic stages. In Stage I, the voltage increases linearly with time, corresponding to the formation of the anodic oxide barrier layer dominated by conventional anodic oxidation. Oxide film formed on the sample surface accompanied by the release of a large amount of oxygen and small quantities of hydrogen and nitrogen, resulting in the formation of the compact gas film. In Stage II, the voltage exhibited a parabolic increase, and fine discharge sparks began to appear on the surface. Driven by the gradient of the electric field intensity, the discharge sparks gradually contracted from the edges toward the center and subsequently distributed uniformly across the entire surface.^30^ The ultrahigh localized temperature generated within the discharge microzones caused partial melting or even vaporization of both the oxide and substrate metal. Upon rapid quenching by the electrolyte, the molten oxides solidified on the substrate surface, forming characteristic crater-like micropores, and the porous ceramic layer started to develop.^31^ In Stage III, the voltage continued to increase slowly with time, while individual discharge sparks became larger and more intense. As the process proceeded, the ceramic layer grew rapidly. Early-formed micropores became interconnected, and some regions were covered by newly molten material, leading to increasingly rough surface morphology.

After completion of the initial MAO process, the power supply was switched to bipolar pulsed mode to conduct S-MAO treatment. During this stage, the negative voltage of all sample groups increased to approximately 90 V, while the positive voltage dropped sharply to the range of 140–170 V. At this stage, the sample surface was covered by the gas film and only weak discharge sparks were observed. The final surface morphologies of the coatings are shown in Fig. 3(c).

The electrolyte with different Zn/Sr ratios leads to slight variations in the anodic voltage-time curves. It is noteworthy that the breakdown voltage is directly related to electrolyte conductivity.^32,33^ As the Sr^2+^ content decreases, the electrolyte conductivity gradually increases (Table 2). Correspondingly, the stable anodic voltage during S-MAO treatment shows a descending trend: Z_1_S_7_MAO > Z_1_S_3_MAO > Z_1_S_1_MAO, with Z_3_S_1_MAO being similar to Z_1_S_1_MAO. Previous studies confirm that a lower anodic voltage effectively suppresses intense discharge events during the MAO process, thereby avoiding the formation of large pores and microcracks induced by high temperatures.^34,35^ It is therefore inferred that the internal stress within the softened spark coating is likely reduced compared to BSMAO.^36^

### 3.2. Phase composition and microstructure of the coatings

**Fig. 4:**
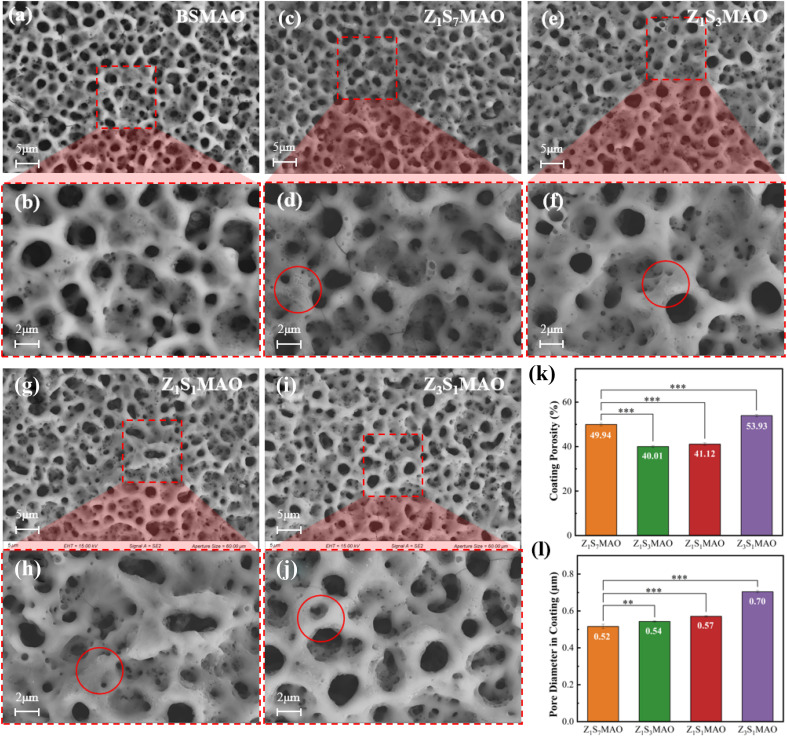
Surface morphologies of (a and b) BSMAO, (c and d) Z_1_S_1_MAO, (e and f) Z_1_S_3_MAO, (g and h) Z_1_S_1_MAO, (i and j) Z_1_S_7_MAO; (k) porosity, (l) average pore size. Significance levels: *: Statistical difference between two corresponding groups connected by horizontal lines, *: *p* < 0.05; **: *p* < 0.01; ***: *p* < 0.001.

The BSMAO treated by MAO exhibited a typical porous structure (Fig. 4(a and b)) with interconnected volcano-shaped morphologies on the surface. These pore structures originate from discharge channels formed by the jetting of molten oxides and gaseous by-products during the MAO process.^37^Fig. 4(c–k) shows the surface morphology of the S-MAO-treated samples obtained at different Sr^2+^/Zn^2+^ ratios. Where small amounts of molten oxide deposits, either granular or powder-like, adhere within or around the discharge pores. Since the softened spark discharge intensity is relatively weak, the surface pore size and distribution of the S-MAO-treated samples show little difference compared to the BSMAO coating. Their pore morphology also does not undergo significant changes. There are not many modifications to the shape of their pores either. This low operating voltage keeps the structural integrity of the first MAO coating, and gentle spark discharges mostly happen in channels that were already there.^20^ Pore density and size statistics (Fig. 4(i–m)) for the S-MAO treated samples show that the Z_3_S_1_MAO sample has the most pores, at 53.90%. Furthermore, the porosity goes down and then back up as the Sr^2+^ content goes down. These little changes are caused by small changes to the surface that happen during the second step of the S-MAO process.

**Fig. 5:**
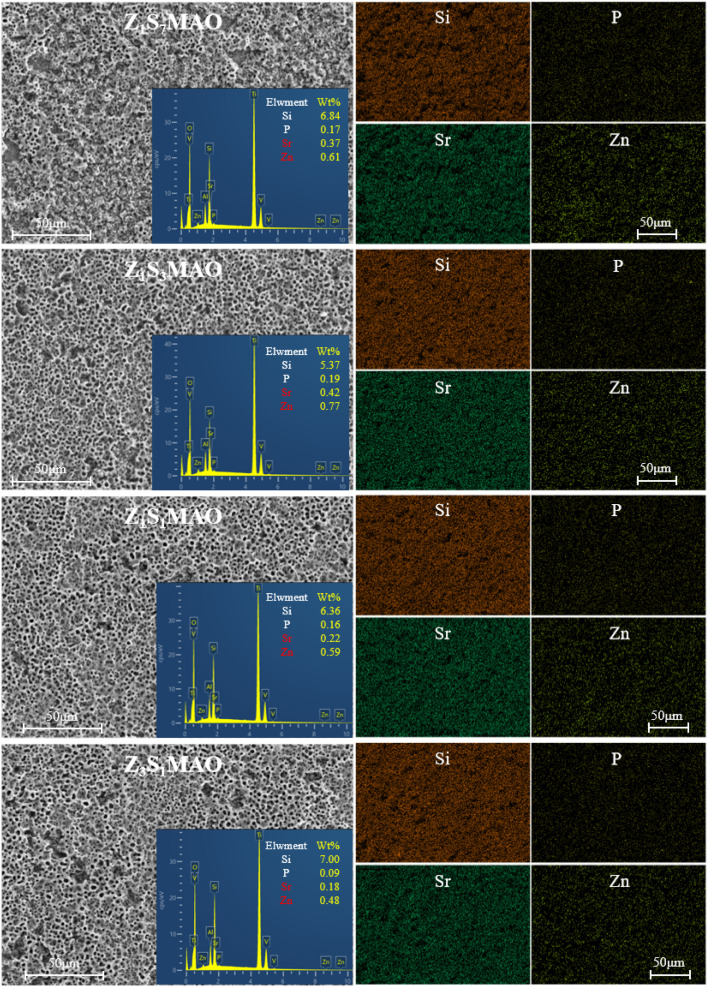
Elemental distribution on the coating surfaces of the processed softened spark MAO titanium alloys obtained from different electrolytes.

Fig. 5 shows the results of the EDS research. The presence of essential elements such as Si, P, Sr, and Zn in all samples validates the effective integration of bioactive components into MAO coatings *via* the two-step method. Si is evenly spread out across the whole coating, with no obvious clumping, and it has a rather high content. On the other hand, P, Sr, and Zn have lower concentrations on the surface.

**Fig. 6:**
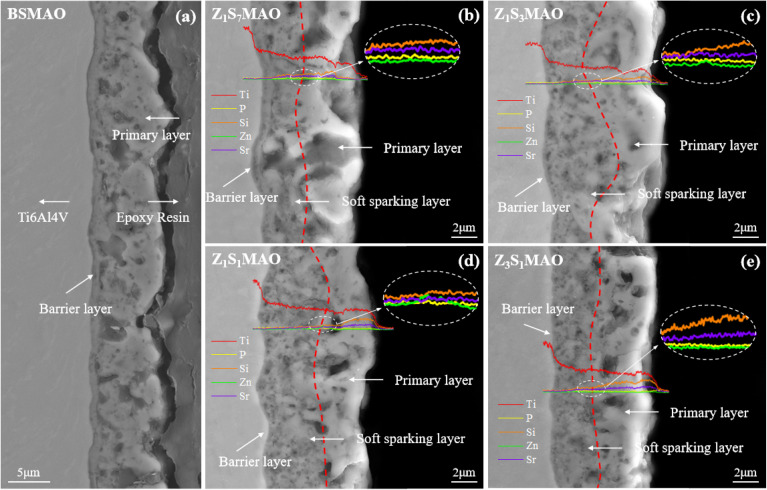
Cross-sectional morphology of the coatings: (a) BSMAO, (b) Z_1_S_1_MAO, (c) Z_1_S_3_MAO, (d) Z_1_S_1_MAO, (e) Z_1_S_7_MAO.

Fig. 6(a) shows cross-sectional morphology of the BSMAO coating. It exhibits bilayer structure, consisting of inner dense barrier layer (∼0.6 µm) and outer porous layer. Total coating thickness is ∼7.5 µm. After S-MAO treatment, overall coating thickness remains comparable to that of BSMAO. However, cross-sectional morphology evolves into distinct three-layer gradient structure (Fig. 6(b–e)). This composite coating comprises inner dense barrier layer, intermediate softened-spark layer, and outer porous layer. Outer porous layer retains some discharge pores (0.5–1 µm in diameter) formed during initial MAO stage. In contrast, softened-spark layer contains numerous finer pores (∼0.1–0.2 µm in diameter) induced by softened spark discharges. Among S-MAO-treated samples, Z_1_S_3_MAO and Z_1_S_1_MAO exhibit relatively uniform softened-spark layer with thickness of ∼4 µm. During S-MAO process, particulate molten oxides generated by softened spark discharges partially fill pores in outer layer or accumulate along pore edges.^36^ This microstructural configuration enhances coating densification while preserving integrity of pre-existing discharge channels. Cross-sectional EDS line-scan analysis further reveals distribution of metallic elements (Sr and Zn). These elements are predominantly enriched in molten oxide regions near outer surface. They also exhibit continuous distribution throughout coating thickness. These results indicate that doped elements are uniformly incorporated during S-MAO process.

**Fig. 7:**
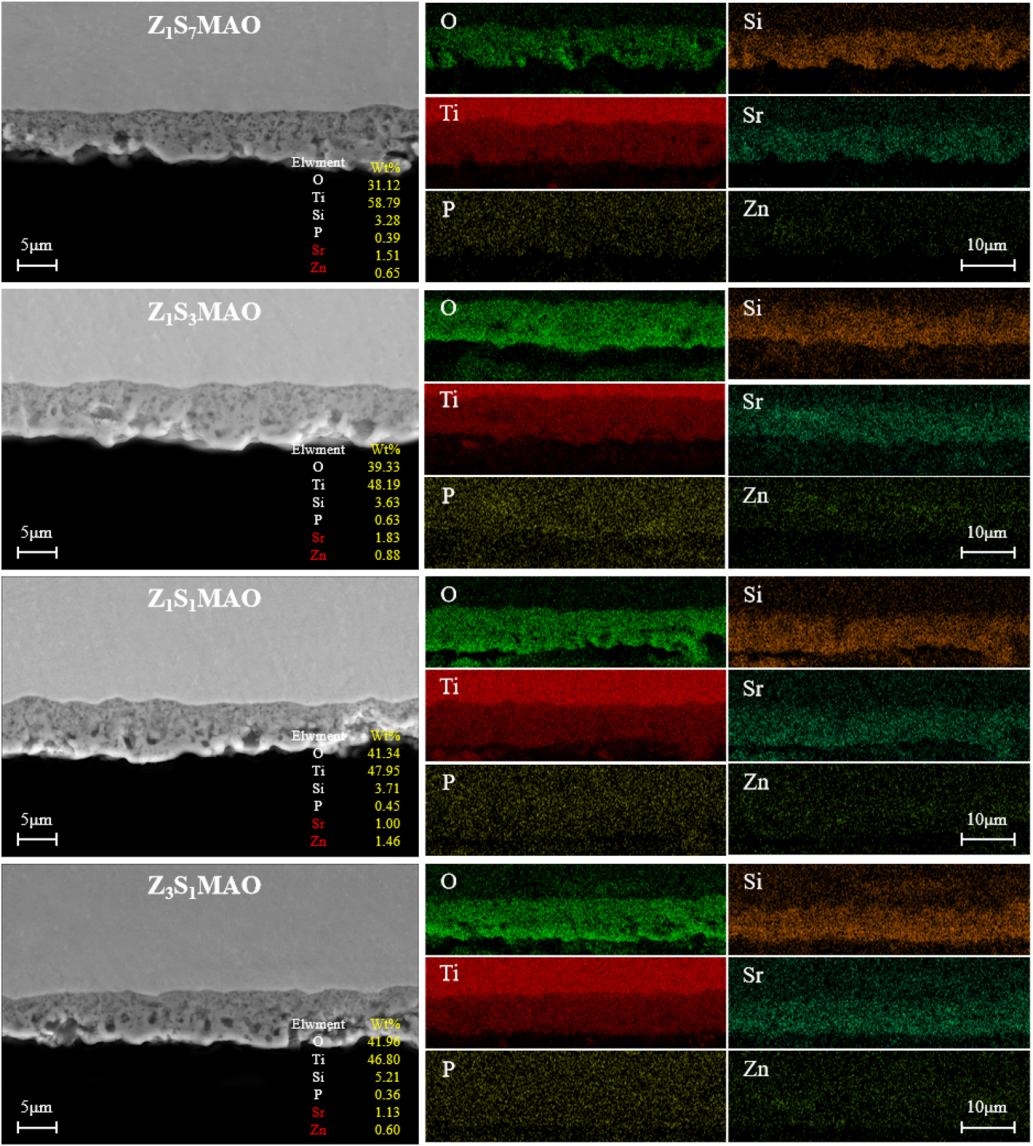
Cross-sectional elemental distribution of the coatings on the processed softened spark MAO titanium alloys prepared in different electrolytes.

Elemental surface scan analysis (Fig. 7) shows that Sr and Zn elements are primarily enriched in internal regions of MAO coating. This indicates effective incorporation of bioactive ions occurs predominantly in sub-surface layer under soft-spark mode. Further line-scan analysis (Fig. 6) reveals that Sr exhibits relatively higher overall deposition, with its concentration gradually decreases from outer porous layer toward inner layers. In contrast, the total deposition of Zn is relatively lower, which is especially evident within the soft-spark region. In coatings of Z_1_S_3_MAO and Z_1_S_1_MAO samples, Zn is distinctly enriched at the interface between softened-spark layer and outer porous layer, forming a characteristic “hump-shaped” distribution. This spatial distribution difference is attributed to relative complexation behaviors of Sr^2+^ and Zn^2+^ in complexing electrolyte.^38^ Studies show that Zn^2+^ typically forms relatively stable complexes with multidentate ligands, while alkaline earth metal ions (such as Sr^2+^) exhibit weaker complexation abilities and higher ligand exchange activities.^39–41^

From this, we can infer that Sr^2+^ ions tend to be in a weakly complexed or largely free state in the high-temperature areas created by S-MAO plasma discharges. In this state, they are more likely to take part in coating development during the soft-spark MAO process. This facilitates their entry into porous regions during early coating formation, resulting in a gradient distribution along the depth direction. In contrast, Zn^2+^ ions tend to undergo localized release and deposition at specific high-energy reaction zones due to their relatively higher complexation stability. This leads to characteristic localized enrichment near the interface between the soft inner layer and outer porous layer. In Z_1_S_7_MAO and Z_3_S_1_MAO samples, distinct Zn enrichment peaks are not observed. This absence may be attributed to the influence of positive voltage during the S-MAO stage, which leads to factors such as non-uniform discharge or insufficient stability of the interfacial reaction zone.

**Fig. 8:**
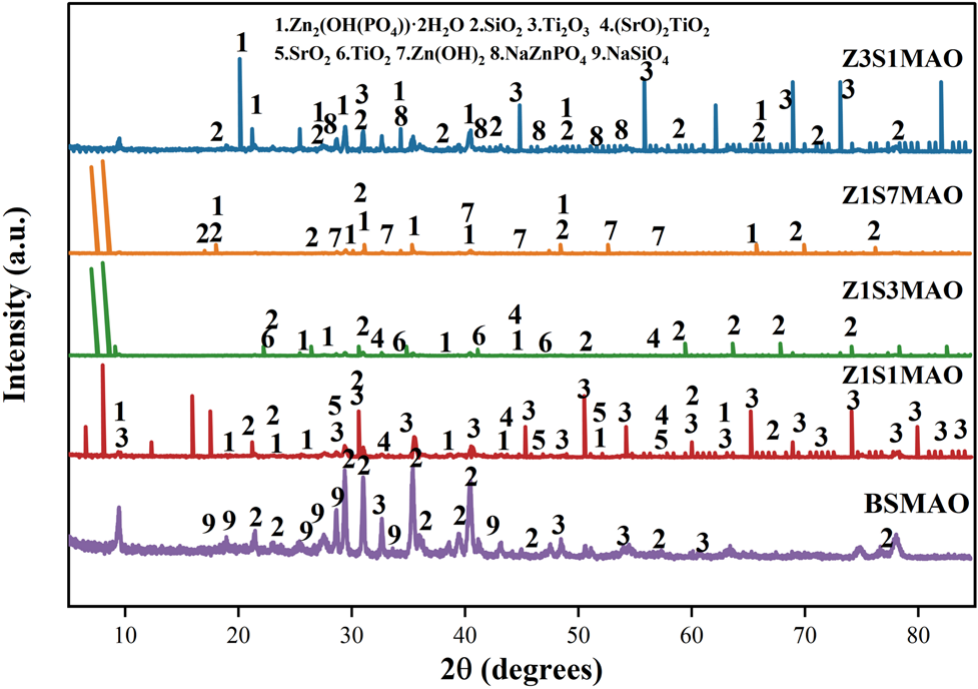
XRD patterns of the coatings on the processed softened spark MAO titanium alloys prepared in different electrolytes.

XRD patterns of the BSMAO coating (Fig. 8) show characteristic diffraction peaks for phases such as SiO_2_ and Ti_2_O_3_. This indicates formation of a composite coating primarily composed of titanium and silicon oxides in the BS electrolyte system. After introduction of the S-MAO process, XRD diffraction peaks become more complex, with changes in peak intensity and width. These changes suggest introduction of additional functional components and regulation of crystallization behavior in the coating. The alternating polarity during softened spark discharge promotes migration and combination of cations and anions, leading to formation of a more complex and multifunctional oxide layer. Diffraction features related to Sr and Zn are detected in all two-step MAO samples. The Sr^2+^/Zn^2+^ ratio significantly influences phase composition and crystallinity of the coating. The Z_1_S_1_MAO sample exhibits relatively high diffraction peak intensity and well-defined peak shapes, indicating higher crystallinity. As Sr content increases (Z_1_S_3_MAO and Z_1_S_7_MAO), diffraction peak intensity gradually decreases. In contrast, increasing Zn content (Z_3_S_1_MAO) facilitates formation of Zn-containing phases, while Sr-containing phases remain detectable.

### 3.3. Electrochemical corrosion performance of MAO coatings

**Fig. 9:**
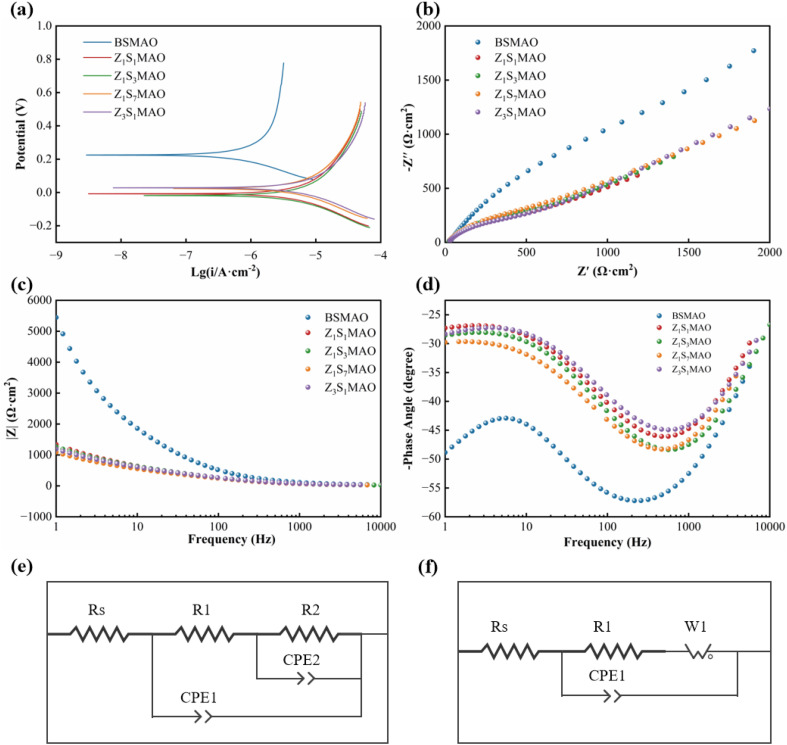
Electrochemical corrosion behavior of different MAO coatings in PBS solution: (a) anodic polarization curves, (b) Nyquist plots, (c) bode |Z| plots, (d) bode phase-angle plots, (e) equivalent circuit model for the MAO coating, (f) equivalent circuit model for the S-MAO coating.

Fig. 9 compares the corrosion resistance of different MAO coatings in PBS solution using anodic polarization curves and electrochemical impedance spectroscopy (EIS). Fig. 9(a) displays the anodic polarization curves, indicating the stability of various coatings during the anodic dissolution phase. All softened spark composite coatings have lower current densities and a positive shift in corrosion potential compared to the BSMAO coating. This means that the composite structure slows down the anodic reaction kinetics to some extent. In addition to morphological data, it may be extrapolated that the MAO coating's highly interconnected pore structure creates continuous permeation routes for the corrosive medium. The softened spark technique, on the other hand, adds a denser inner layer at the interface between the coating and the substrate. This makes the negative influence of pore connectivity on the corrosion process less strong.^42^

The Nyquist plot (Fig. 9(b)) shows that BSMAO is very different from the ideal semicircular capacitive arc. This is because it diffuses slowly at low frequencies, which implies that both charge transfer and diffusion impedance control its corrosion process at the same time. This is because it has a porous, loose structure and pores that are very well connected. The softened spark composite coatings, on the other hand, display more equal capacitive arcs and a lot less low-frequency diffusion. This means that the interface structure is more even and the electrochemical response is more stable. The Z_1_S_3_MAO and Z_3_S_1_MAO samples have larger capacitive arc radii, which means that the impedance of charge transfer at their interface is substantially higher. This makes the electrochemical protection for the substrate work better.^43^

The Bode |Z| and phase-angle plots (Fig. 9(c–d)) further corroborate these trends. Although the BSMAO coating exhibits a relatively high |Z| (∼2.4 × 10^5^ Ω cm^2^ at 0.01 Hz, Table 3) value in the low-frequency region, this response mainly originates from the temporary hindrance of ion diffusion by its loose outer porous layer. Once corrosive species penetrate through interconnected pores and establish continuous pathways, the protective capability of the coating rapidly deteriorates. In contrast, the softened-spark composite coatings maintain a more stable low-frequency impedance response and exhibit a single, broadened phase-angle peak. It indicating that their corrosion process is predominantly governed by interfacial charge transfer, which implies more reliable long-term corrosion protection.^44^

**Table 3:** Electrochemical fitted parameters of the initial MAO coating

Sampels	Rs (Ω cm^2^)	R1 (Ω cm^2^)	R2 (Ω cm^2^)	CPE2-T (Ω^−1^ S^n^·cm^−2^)	CPE2-P (Ω^−1^ S^n^·cm^−2^)	CPE1-T (Ω^−1^ S^n^·cm^−2^)	CPE1-P (Ω^−1^ S^n^·cm^−2^)
BSMAO	26.49	3.03 × 10^4^	2.4045 × 10^5^	4.7479 × 10^−5^	0.6861	1.5653 × 10^−5^	0.7383

The influence of coating structure on corrosion behavior is further quantified by equivalent circuit fitting. The BSMAO coating can be well described by the Rs–(R1∥CPE1)–(R2∥CPE2) model (Fig. 9(e)), where the additional R2–CPE2 time constant corresponds to the dense inner barrier layer, contributing a high-resistance response (Table 3). In contrast, all softened-spark MAO coatings are adequately fitted using the Rs–(R1∥CPE1)–W1 model (Fig. 9(f)), indicating that their electrochemical response is dominated by a single interfacial time constant coupled with diffusion-related impedance. The absence of an additional R2–CPE2 element suggests that the softened-spark process does not introduce a new independent electrochemical layer but instead modifies the pore structure and enhances interfacial compactness.

It should be stressed that, due to the differing equivalent circuit models employed, the physical significance of the R1 parameter in BSMAO and S-MAO coatings is not directly comparable. For the BSMAO coating, the interfacial charge-transfer process is predominantly represented by the R2 component, but in softened-spark MAO coatings, the corrosion process can be sufficiently described by a single interfacial charge-transfer resistance. Therefore, the improved R1 values seen with softened-spark coatings represent enhanced interfacial compactness and stability rather than a mere numerical increase.

In the S-MAO system, the interfacial charge-transfer resistance R1 rises when the Sr/Zn ratio is optimized, and the effect of Warburg diffusion impedance slowly fades (Table 4). And showing that ion transport through linked pores is effectively blocked and that corrosion behavior changes from being governed by diffusion to being dominated by interfacial charge transfer.^45^ The Z_1_S_3_MAO coating (Sr/Zn = 3 : 1) had the highest R1 value (443.6 Ω cm^2^) of all the samples. This means that it has the most compact interfacial structure and is the best at resisting corrosion.

**Table 4:** Electrochemical fitted parameters of the softened spark MAO coatings

Samples	Rs (Ω cm^2^)	R1 (Ω cm^2^)	W1-R (Ω cm^2^)	W1-T (Ω cm^2^)	W1-P (Ω cm^2^)	CPE1-T (Ω^−1^ S^n^ cm^−2^)	CPE1-P (Ω^−1^ S^n^ cm^−2^)
Z_1_S_7_MAO	21.27	394.7	83.18	2.71 × 10^−4^	0.1882	2.636 × 10^−5^	0.7206
Z_1_S_3_MAO	17.20	443.6	1.21 × 10^4^	97.72	0.3893	2.5471 × 10^−5^	0.7257
Z_1_S_1_MAO	18.29	413.0	6.13 × 10^3^	14.11	0.3791	2.6562 × 10^−5^	0.7314
Z_3_S_1_MAO	18.67	427.1	7.39 × 10^3^	35.4	0.4050	3.3497 × 10^−5^	0.6945

### 3.4 Hydrophilicity and biological performance of the composite coatings

**Fig. 10:**
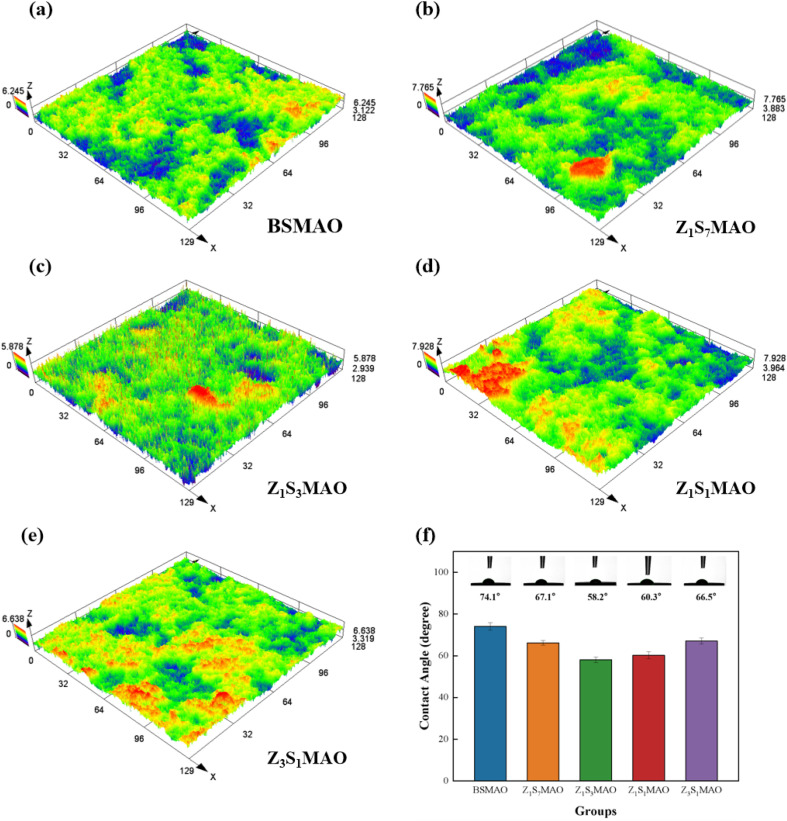
3D surface topography: (a)BSMAO, (b)Z_1_S_1_MAO, (c)Z_1_S_3_MAO, (d)Z_1_S_1_MAO, (e)Z_1_S_7_MAO; (f) water contact angles of the coatings.

The surface wettability of different MAO coatings was evaluated by water contact angle measurements. As shown in Fig. 10(f), all samples exhibit contact angles below 90°, indicating that both the initial MAO coating and the Sr/Zn co-doped composite coatings possess overall hydrophilic characteristics. Compared with the single-layer BSMAO coating (74.1° ± 2.8), all composite coatings treated by S-MAO show markedly reduced contact angles (58.2° – 66.5°), suggesting a correlation between the surface structures formed during the secondary treatment and the enhanced wettability. Among them, the Z_1_S_3_MAO and Z_1_S_1_MAO samples display relatively low and comparable contact angles.

To further elucidate the surface characteristics, three-dimensional surface morphologies of the coatings were analyzed using laser confocal microscopy (Fig. 10(a–e)), and the corresponding roughness parameters are summarized in Table 5. The BSMAO coating exhibits relatively smooth surface undulations, with the lowest Sq (0.478 µm) and Sa (0.372 µm), consistent with its comparatively higher contact angle. The softened spark-treated samples show an overall increase in surface roughness, with Z_1_S_3_MAO presenting the highest Sq value (0.517 µm), indicating more pronounced micro-scale surface fluctuations. In addition, all samples exhibit negative surface skewness values (Ssk = −0.291 ∼ −0.485), suggesting that pit-dominated surface features prevail, which is consistent with the formation of discharge channels and pores during the MAO process. It should be noted that the variation in wettability cannot be attributed to a single roughness parameter alone, but is more likely associated with the synergistic effects of surface micro-scale undulations and pore morphology. In this regard, the Z_1_S_3_MAO coating exhibits a more balanced combination of contact angle, surface topography, and pore distribution.

**Table 5:** Surface roughness of the MAO coatings

Samples	Sq (µm)	Sku	Sv (µm)	Sa (µm)	Ssk	Sp (µm)	Sz (µm)
BSMAO	0.478	3.734	3.217	0.372	−0.291	3.079	6.296
Z_1_S_7_MAO	0.489	4.172	3.072	0.374	−0.067	4.038	7.11
Z_1_S_3_MAO	0.517	4.308	3.881	0.397	−0.359	3.717	7.598
Z_1_S_1_MAO	0.501	4.171	3.682	0.384	−0.298	3.388	7.07
Z_3_S_1_MAO	0.484	4.041	3.271	0.374	−0.485	2.755	6.026

**Fig. 11:**
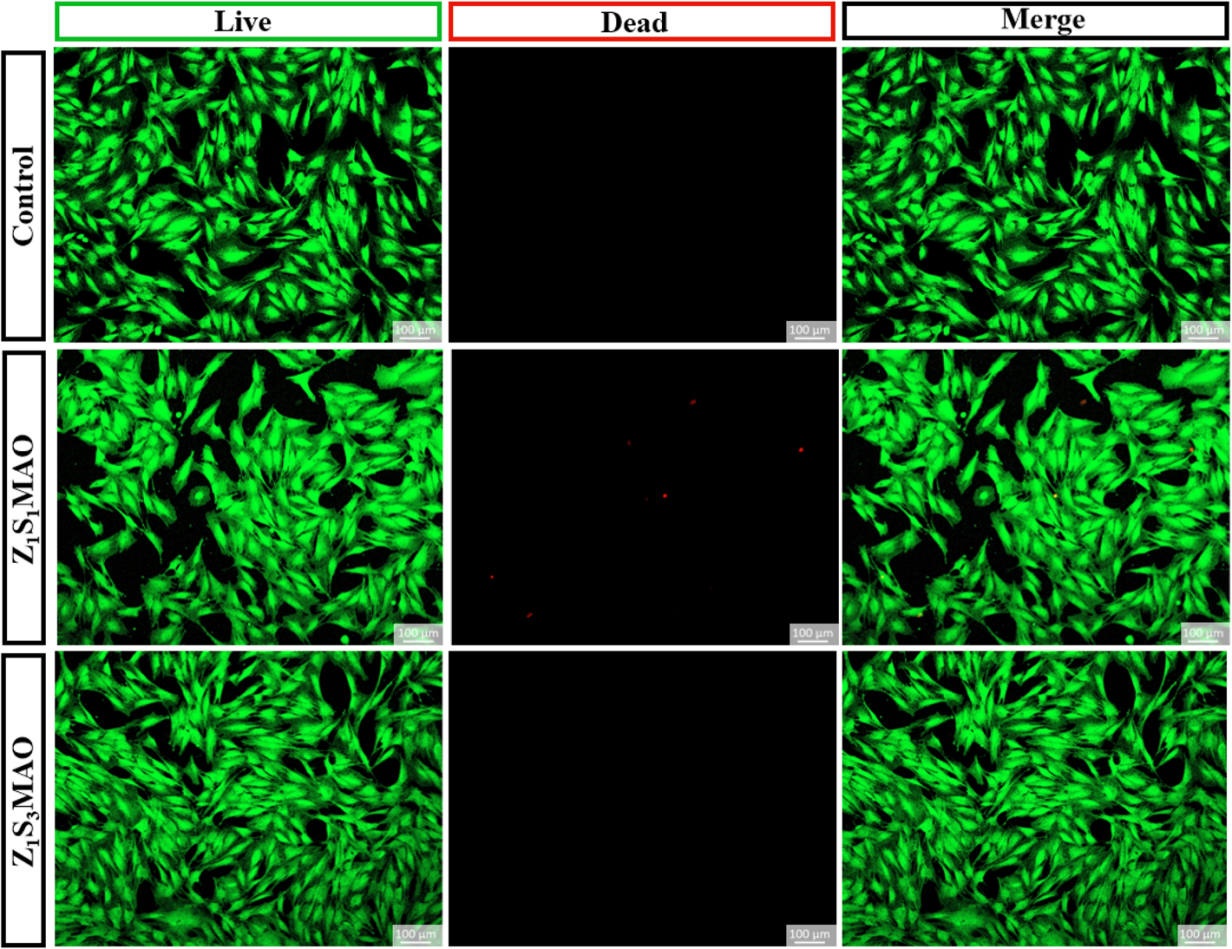
Viability of MC3T3-E1 cells on Z_1_S_1_MAO and Z_1_S_3_MAO scaffolds after 24 h of culture (green: live cells; red: dead cells; calcein-AM/PI staining).

Biocompatibility is an important evaluation criterion for bone scaffold materials and is closely associated with the ability of the material surface to support cell adhesion and survival. In this study, the *in vitro* cytocompatibility of the blank control, Z_1_S_1_MAO, and Z_1_S_3_MAO samples was preliminarily evaluated using LIVE/DEAD fluorescence staining (Calcein-AM/PI dual staining). As shown in Fig. 11, cells in the blank control group exhibited typical spindle-shaped or polygonal morphologies with extensive spreading, and interconnected filopodia formed a continuous cellular network, indicating good adhesion behavior. Compared with the control, cells cultured on the Z_1_S_1_MAO surface remained predominantly spindle-shaped; however, a slight reduction in filopodia extension and spreading area was observed, accompanied by a small number of scattered PI-positive signals in localized regions. Nevertheless, the majority of cells maintained intact morphologies without obvious deformation, suggesting that the coating with an Sr/Zn ratio of 1 : 1 provides an overall acceptable surface environment for cell survival. In contrast, cells on the Z_1_S_3_MAO surface exhibited morphologies more similar to those of the control group, with well-developed filopodia, extensive spreading, and fewer PI-positive signals, indicating a relatively higher level of cell viability. Overall, among the investigated samples, the Z_1_S_3_MAO surface with a higher Sr/Zn ratio demonstrates comparatively more favorable characteristics in terms of maintaining cell morphology and supporting cell survival.

## 4. Conclusions

This study successfully constructed a composite bioceramic coating on Ti6Al4V dental implants by optimizing the Zn/Sr ion ratio in the electrolyte, combined with MAO and S-MAO. The results indicate:

(1). By optimizing the Zn/Sr ion ratio (1 : 3 and 1 : 1) and combining a two-step MAO process, composite coatings with a “barrier layer/softened-spark layer/porous layer” gradient structure were successfully fabricated. This process, achieved through stable discharge voltages (145–160 V), promotes a uniform and gentle discharge process, resulting in a structurally complete and evenly thickened softened-spark layer. This effectively eliminates the connected pore defects in conventional coatings and significantly enhances the corrosion resistance of Ti6Al4V.

(2). S-MAO, while preserving the inherent porous microstructure of the coating, achieved effective multi-element doping. Sr^2+^ predominantly accumulates in the outer porous region, while Zn^2+^ forms a characteristic “hump-like” enrichment zone at the interface of the softened-spark layer, creating an ion-release microzone. When the Zn/Sr ratio is 1 : 3, the composite coating exhibited improved biological functionality and electrochemical stability.

This study provides theoretical foundation for developing advanced corrosion-resistant surface coatings for dental implants. Future research can further explore the synergistic doping effects of other bioactive elements.

## Author contributions

Y. H.: conceptualization, methodology, writing–original draft; writing – review & editing. L. N.: data curation, funding acquisition, writing – review & editing. J. F.: formal analysis, resources, supervision, writing – review & editing. X. W.: investigation, funding acquisition, writing – review & editing. Z. Z.: software, validation, writing – review & editing. All authors reviewed and approved the final manuscript.

## Conflicts of interest

The authors declared no conflict of interest.

## Data Availability

The authors confirm that the data supporting the findings of this study are available within the article.
